# Economic policy uncertainty and corporate innovation-empirical evidence from Chinese pharmaceutical listed companies

**DOI:** 10.3389/fpubh.2024.1411495

**Published:** 2025-01-03

**Authors:** Zikun Wu, Su Wang, Licong Kuang, Yuwen Chen

**Affiliations:** ^1^School of Business Administration, Shenyang Pharmaceutical University, Shenyang, China; ^2^Betta Pharmaceuticals Co., Ltd, Hangzhou, China; ^3^Drug Regulatory Research Base of NMPA, Research Institute of Drug Regulatory Science, Shenyang Pharmaceutical University, Shenyang, China

**Keywords:** economic policy uncertainty, corporate innovation, financialization, listed pharmaceutical companies, cost

## Abstract

**Introduction:**

Under the background that economic policy uncertainty tends to be normal, the innovation behavior of enterprises can cope with the cost impact brought by economic policy uncertainty.

**Methods:**

Based on the relevant data of China’s A-share pharmaceutical listed companies from 2015 to 2022, this paper empirically studied the relationship between economic policy uncertainty and firm innovation by using fixed-effect model, intermediary model, instrumental variable method and two-step method, and investigated the mechanism effects of financialization, executive compensation and government subsidies.

**Conclusion:**

Economic policy uncertainty significantly increases the innovation intensity of enterprises. Enterprises with dual management, overseas background and non-state-owned ownership will increase their innovative behavior in the face of economic policy uncertainty. Further analysis shows that economic policy uncertainty increases the innovation intensity of enterprises through three mechanisms: increasing financial transformation, obtaining government subsidies and motivating management. Increasing the innovation intensity of enterprises can deal with the risk impact caused by economic policy uncertainty, but can not deal with the negative impact of cost increase. The transformation of financialization can help enterprises cope with the operational risks and later costs caused by economic policy uncertainty.

**Discussion:**

This study provides empirical evidence for the theoretical inference that firms respond to the impact of economic policy uncertainty and thus increase their innovation behavior.

## 1 Introduction

In recent years, the external business environment of various enterprises has been characterized by instability, largely as a consequence of the impact of the global financial crisis, depressed international markets, low consumer dynamics, and public safety and health incidents. A comparison of the economic policy uncertainty index, as measured by Scott Baker, reveals that China’s economic policy uncertainty index in 2022 is 12.8 times higher than that of 2000.^1^ Additionally, the team assesses the economic policy uncertainty of other countries. A comparison of the data reveals that the U.S. economic policy uncertainty index in 2022 is 1.17 times higher than that of 2000, while the world economic uncertainty index in 2022 is 3.78 times that of 2000. The gradual increase in economic policy uncertainty has had a detrimental impact on the normal functioning of the economy. This has manifested in various ways, including a negative effect on stock market returns (1, 61), a deterioration in market expectations (2), an exacerbation of capital outflows, and the triggering of economic turmoil in a number of countries, which has led to increased volatility in the food market (3). In order to circumvent the repercussions of the economic crisis, countries implement irregular adjustments to their economic policies in order to navigate the market turbulence, which in turn gives rise to an increase in the economic policy uncertainty index. In the context of rising economic policy uncertainty, the question of how enterprises can build their competitive advantage to cope with the various adverse effects has become a key research topic. Innovation is widely recognized as a driving force of development, and the ability to innovate is crucial to the sustainable development of enterprises. However, the continuous changes in economic development policies make the development environment of enterprises dramatically turbulent, which impacts the enterprises’ R&D and innovation activities. The manner in which economic policy uncertainty affects enterprise behavior has become a topic of growing interest among scholars, who are engaged in research to elucidate the pathways of influence.

In previous studies, research on economic policy uncertainty has focused on economic activities such as trade and import/export, business transformation, and unemployment, while after the outbreak of the COVID-19 pandemic in 2020, economic policy uncertainty has increased dramatically worldwide, and the volatility of the macroeconomic environment has caused firms’ strategic planning and resource allocation to be affected. The research focuses on how economic policy uncertainty affects corporate finance, capital flows, and investment, among others, in order to counteract the shocks caused by economic policy uncertainty. Enterprise innovation is the main way for manufacturing enterprises to develop their core business and enhance their competitiveness, but enterprise innovation is also accompanied by long cycles, high investments, and irreversibility, which is more obvious in the pharmaceutical field. With the increasing trend of aging and changes in the disease spectrum, products in the pharmaceutical manufacturing industry need to be constantly updated and iterated, and enterprises need to continuously invest in innovation to improve their competitiveness. To maintain competitiveness and market share, pharmaceutical companies must constantly seek new areas of growth. Innovation has become a key way for companies to manage uncertainty and achieve sustainable development. When the external economic environment is turbulent, companies will choose to tighten their capital, save money in all aspects of spending, and prioritize the allocation of funds to more promising projects, which will have an impact on the company’s competitiveness.

For enterprises, on the one hand, economic policy uncertainty will promote enterprises to enhance their sense of crisis, actively save cash flow, avoid unnecessary expenses, and enhance innovation investment in their main business in order to increase their competitiveness; on the other hand, economic policy uncertainty will increase the difficulty of financing for enterprises, leading to an increase in enterprise risk, and enterprises will reduce their investment and innovation activities in order to avoid the risk. Therefore there are two views on economic policy uncertainty, one view is that firms’ innovation activity is a continuous investment activity that requires high adjustment costs, and uncertainty can increase firms’ resilience by actively saving cash in the early stage, making it possible to have enough funds to maintain R&D activities in uncertain times, so as to stay ahead of competing firms and gain an over-expected market share. In the case of economic policy uncertainty, pharmaceutical enterprises find it difficult to accurately predict the direction and impact of the policy, there may be huge risks, but due to the long cycle of pharmaceutical product innovation, the characteristics of continuous investment, pharmaceutical enterprises still need to maintain a continuous investment in technological innovation, through increasing R&D efforts, the introduction of advanced technology and management experience, enterprises can continue to enhance their innovation capabilities and core competitiveness to better cope with market changes and competitive challenges. The other view is that economic policy uncertainty makes it difficult for firms to meet their annual expectations, leading to fluctuations in firms’ market expectations and inhibiting their innovative activities, which will lead to serious operational risks, reduced capital expenditure, and higher financing costs for firms. Economic policies uncertain policies may have an impact on market supply and demand, market prices, and the competitive landscape of the market, and pharmaceutical companies are often influenced by market expectations when making technology innovation decisions. Uncertain market expectations may make pharmaceutical manufacturing enterprises more cautious in technological innovation, thus affecting innovation effectiveness and market competitiveness. As a result of increased financing costs and risks, pharmaceutical manufacturing firms may make decisions to reduce investment in innovation.

One potential solution to the issue of financing difficulties in the process of financialization, which can be defined as the structural optimization of firms in order to mitigate the impact of external shocks. In the context of economic uncertainty, enterprises are compelled to adapt their internal strategies and structures in order to mitigate the impact of external environmental factors. The potential risks associated with production and operational challenges disrupted capital chains, and financing constraints provide enterprises with a compelling motivation to expedite the transformation of financialization. On the one hand, enterprises leverage the reservoir effect of financialization to accumulate and enhance their asset liquidity and liquid cash reserves, thereby reducing the capital breakage of enterprise-related activities (4, 5); alternatively, from a financing perspective, enhanced transparency of corporate information and increased financial investment can mitigate the risk of collapse (6) and alleviate constraints on financing (7). An increase in financial investment can alleviate constraints on financing and prompt enterprises to allocate more funds to the R&D process. The continuity of R&D funding enables enterprises to enhance their innovation capabilities, strengthen their product competitiveness, and improve their resilience to external environmental risks. This illustrates the efficiency of financialization in mitigating the impact of business risks associated with economic policy uncertainty. Pharmaceutical companies, due to the distinctive characteristics of the industry, require sustained innovation to bolster enterprise competitiveness. Consequently, the demand for innovation is particularly high in this sector.

In addition to structural optimization, internal management optimization is also a strategy employed by firms to mitigate the impact of external shocks. Executive incentives represent a specific form of internal management optimization pursued by firms to enhance their resilience to external transformations. Prior research has demonstrated that executive incentives constitute a significant factor influencing managerial decisions to increase investment in corporate innovation. In the context of economic policy uncertainty, firms are exposed to a dual risk: that of their main business and that of changes in their own managers. On the one hand, executive compensation incentives can effectively address the principal-agent conflict, mitigate the risk-aversion tendency of management, and stimulate enthusiasm for venture capital projects, thereby increasing the innovation output of the enterprise (63). Conversely, executive compensation incentives can be viewed as a cost-effective internal governance mechanism. They have the potential to curtail the exploitative practices of senior managers with regard to resources, whilst simultaneously enhancing the efficiency of R&D activities (9). By implementing reasonable improvements to the executive incentive policy, it is more feasible to stabilize the internal governance of the firm and provide guidance against external risk shocks.

In the market mechanism, the enterprise’s R&D and innovation activities are external, and the enterprise has to bear all the R&D costs in the R&D and innovation activities, in the background of the economic policy uncertainty, the impact brought by the external environment may make the enterprise to reduce the income that it should have, so as to reduce the enterprise’s R&D enthusiasm. In this context, government subsidies will help enterprises get rid of financial difficulties and better deploy resources to innovative R&D (10). On the one hand, pharmaceutical enterprises are technology-driven, and such enterprises must provide sufficient funds for research to develop new products or services (11) to ensure the competitiveness of their own business, and government subsidies offset the financial constraints caused by market failures by compensating for innovation inputs (12); on the other hand, targeted government subsidies can help redistribute the labor force (8) and enhance the innovation efficiency of enterprises.

In light of the aforementioned analyzes, the objective of this research is to examine the impact of economic uncertainty on innovation in the pharmaceutical industry. To this end, the following questions will be addressed: firstly, how does economic policy uncertainty affect innovation in pharmaceutical manufacturing firms? Secondly, it is necessary to identify the mechanism through which economic policy uncertainty affects innovation in pharmaceutical manufacturing companies. Thirdly, what strategies can be employed to mitigate the impact of economic policy uncertainty fluctuations?

In order to respond to the aforementioned questions, this paper utilizes data from China’s A-share listed pharmaceutical manufacturing firms from 2015 to 2022, with the objective of evaluating the influence of economic policy uncertainty on pharmaceutical manufacturing firms’ innovation. This paper makes a number of contributions to the existing literature. Firstly, it explores the factors affecting corporate innovation from the perspective of economic uncertainty. While existing studies have explored the factors affecting corporate innovation from the perspective of policy incentives based on government subsidies (13), corporate governance (14), and internationalization (15), this paper explores the factors affecting corporate innovation from the perspective of macroeconomic uncertainty. This leads to the rise of economic policy uncertainty, which increases the willingness to innovate in response to external shocks, further expanding the study of corporate innovation from a macro perspective. This paper contributes to the existing literature on enterprise innovation by examining the willingness of enterprises to innovate in response to external shocks due to rising economic policy uncertainty from a macro perspective. Secondly, the mechanism of action is subjected to further examination. The extant literature on economic policy uncertainty has primarily focused on examining the impact of such uncertainty on investment risks, stock returns, and the financial industry as a whole. Additionally, studies on the relationship between economic policy uncertainty and innovation have predominantly concentrated on the industry as a whole or the manufacturing industry, with a comparatively limited number of studies examining the pharmaceutical manufacturing industry. In the context of the current era, which is characterized by unprecedented change, economic policy uncertainty is likely to persist for the foreseeable future. The pharmaceutical manufacturing industry is witnessing the emergence of an increasing number of innovative drugs, and economic policy plays an important role in guiding the strategic direction of enterprises. Consequently, it is becoming an increasingly influential factor. This paper examines the role of the enterprise in the innovation process from both internal and external perspectives. It confirms that reasonable financialization, executive incentives, and government subsidies can effectively enhance the enterprise’s innovation ability, improve the enterprise’s own ability to cope with the impact of economic policy uncertainty, and provide empirical evidence for the enterprise to enhance its innovation ability. Thirdly, the present study focuses on the pharmaceutical manufacturing industry. Currently, there is a paucity of research examining the nexus between economic policy uncertainty and enterprise innovation. The majority of extant studies tend to adopt a macro-level perspective, with scant attention paid to the pharmaceutical industry. In the current era, the pharmaceutical manufacturing industry has garnered considerable attention, particularly in light of the promotion of the strategy of Healthy China. The advent of innovative drug companies has underscored the necessity for pharmaceutical enterprises to prioritize innovation as a cornerstone of their organizational development. This paper will examine the influence of research and development (R&D) and innovation within the pharmaceutical industry through the lens of economic policy uncertainty, thereby addressing existing gaps in the literature and providing crucial insights for effectively navigating the impact of economic policy uncertainty on pharmaceutical manufacturing enterprises and enhancing their self-competitiveness. It offers a crucial point of reference for pharmaceutical manufacturing enterprises seeking to mitigate the impact of economic policy uncertainty and enhance their competitiveness. The contemporary global context is characterized by complexity and volatility, with the ascendance of uncertainty representing a pervasive challenge for humanity. This paper illustrates that, in the context of economic policy uncertainty, the utilization of appropriate tools is essential for enhancing enterprise resilience to external shocks and facilitating sustainable development.

This paper is divided into five sections, Section 1 is the introductory section; Section 2 is a summary of the relevant literature on economic policy uncertainty and the formulation of the research hypotheses; Sections 3–5 are the modeling and data analysis; Section 6 is the conclusions and recommendations; and Section 7 is the shortcomings of the article.

## 2 Literature review and research hypotheses

### 2.1 Economic policy uncertainty and firms’ R&D intensity

The concept of economic policy uncertainty can be defined as the inability of economic agents to accurately predict the timing, nature and extent of future changes to the government’s current economic policy (16). For an extended period, scholars have engaged in the study of economic operations and market regulations. In the wake of the global economic crisis of 2008, countries have intervened in the real economy and modified economic policy on an ongoing basis in order to mitigate the risk of another crisis. However, this has led to an increase in economic policy uncertainty. China has been undergoing a critical period of economic development, progressing from a high-speed to a high-quality growth trajectory. This has entailed frequent adjustments to reform policies across a range of sectors, including finance, property, manufacturing and capital markets. While these measures have had discernible consequences, they have also heightened uncertainty. From an economic perspective, policy uncertainty has a significant impact on a number of key areas, including macroeconomic operations, monetary policy, and corporate behavior. In a study conducted in 2017, Knut Are Aastveit employed structural vector autoregression to examine the interaction between economic policy uncertainty and monetary policy shocks in the United States (17). The findings indicated that when uncertainty is elevated, the impact on investment behavior is particularly pronounced. In a similar study conducted in 2019, He Minyuan and colleagues observed that when economic policy uncertainty is higher, the tax burden on firms is heavier (18). Zheng et al. (2020) demonstrated that economic policy uncertainty has a detrimental impact on firms’ innovation behavior. Conversely, economic policy uncertainty can affect the internal operations of firms. Sha et al. (19) investigated the relationship between economic policy uncertainty and mergers and acquisitions in China. Their findings suggest that Chinese firms are more likely to engage in acquisitions and increase the wealth of the acquirer during periods of high economic policy uncertainty. In a study conducted in 2019, Chen discovered a negative correlation between economic policy uncertainty and corporate risk-taking, among other factors (27). Zhang (20) and Qi et al. (21) demonstrated that economic policy uncertainty exerts an influence on investor sentiment and financial stability.

As noted by Nick (66), frequent alterations to macroeconomic policy can result in transient negative impacts on firms during periods of economic contraction. In response, firms may transition from short-term to long-term investments, aiming to secure more resilient economic returns. In accordance with the tenets of the growth option theory, it can be posited that when confronted with the specter of economic policy uncertainty, the prospective advantages of a firm’s innovative conduct may be regarded as a growth option. It is further postulated that the ascendant uncertainty will engender a greater propensity to utilize the option, thereby enhancing the anticipated returns (22). It has been demonstrated that economic policy uncertainty engenders augmented profits and enhances the option value of the firm (23). As the value of the enterprise’s options increases, so too does the benefit derived from innovation output, prompting the enterprise to increase the intensity of its innovation investment and thereby creating a positive feedback loop. The analysis of competitive analysis theory indicates that economic policy uncertainty will increase the information asymmetry of the enterprise, resulting in higher costs. However, the enterprise will seek to enhance its competitiveness and operational efficiency by increasing its innovation investment and market share (24, 25). In light of the aforementioned theory, the following hypothesis is put forth:

*H1:* An increase in economic policy uncertainty has been observed to result in a heightened level of investment in innovation by firms.

### 2.2 Mechanisms by which economic policy uncertainty affects innovation intensity

#### 2.2.1 Financialization of enterprises

The fundamental characteristics of continuous innovation are a lengthy cycle time, elevated risk, and a robust demand for capital. This implies that organizations must sustain their capital investments to maintain uninterrupted innovation and exhibit a heightened need for liquidity. It is not uncommon for non-financial firms to engage in financialization, which can be regarded as a normal investment activity that enables firms to pursue market profits and support their innovative behavior. Lee et al. (26) found that non-financial firms are motivated to hold financial assets mainly due to precautionary motives and to hold moderate holdings of financial assets against liquidity risk. The volatility of economic policies gives rise to an increase in market risk. In order to mitigate financing constraints and hedge risks, non-financial firms will hold more liquid financial assets for precautionary motives. By holding financial assets, firms can enhance their cash liquidity and ensure sufficient internal funding, while also mitigating the pressure and dependence on external financing. Hu et al. (7) posited that corporate financialization can satisfy the requirements of corporate innovation funds, thereby initiating innovative activities and enhancing corporate competitiveness. In the context of economic policy uncertainty, firms face heightened pressure to secure external financing, underscoring the need for sufficient cash reserves to support innovation. Corporate financialization serves as a reservoir, increasing corporate financial assets to satisfy corporate innovation activities, ensure innovation sustainability, and withstand negative shocks from economic policy uncertainty (59, 62). Concurrently, Jia (67) and other scholars posit that organizations should contemplate the prospective operational challenges and financial crises that they may encounter in the future. They advocate for an increase in liquid assets, such as financial assets that can be readily realized. In the event of insufficient funds for investment, production, and operational activities, and the capacity to provide financial support for continuous technological innovation, the enterprise may consider enhancing its innovation investment (28). This leads to the formulation of hypothesis two:

*H2:* Economic policy uncertainty has a positive effect on firms’ innovation inputs through the financialization mechanism.

#### 2.2.2 Executive motivation

The R&D and innovation activities of enterprises are influenced by a range of internal and external factors. In addition to these external factors, managers represent another internal factor that affects the activities of enterprises (29). In accordance with the principal-agent theory, executives, in their capacity as agents of the enterprise, are driven by a combination of their own interests and those of the enterprise to pursue excess investment returns from the market (30). In accordance with the theory of high management, the actions of executives themselves can influence the enterprise’s decision-making processes (31). It is inevitable that managers will be biased based on their interests. In selecting strategies for the short term, managers will favour those that benefit their interests, and will tend to avoid risky decisions such as high-risk research and development innovations (32). With regard to executive compensation, Zhou and Yang (33) posits that salary incentives for executives can foster convergence between personal and corporate interests, thereby enhancing their willingness to innovate and motivation to develop. Lu and Liang (34) and others contend that executive compensation incentives can improve corporate performance and significantly enhance the innovative performance of the enterprise, thereby improving business innovation. Dong and Qin (35) further argues that executive compensation incentives can promote innovation programs and the transformation of innovation results. It is thought that increased executive compensation incentives will produce an “innovation compensation effect,” which will motivate executives to devote more time and energy to innovation management and reduce the tendency to avoid certain risky decisions. In the context of economic policy uncertainty, it is possible that executives may opt for more stable options. In the face of environmental uncertainty, employees may be driven to change jobs more frequently, thereby introducing significant internal instability into the enterprise. Moreover, an increase in executive incentive behavior may encourage executives to prioritise the company’s innovative projects. When firms give more incentives to managers, it will encourage managers to care more about the long-term development of the firm, and it will also mean that firms will provide more attention and support to innovation activities, leading to hypothesis three in this analysis:

*H3:* The uncertainty of economic policy has been demonstrated to stimulate corporate investment in innovation by enhancing the incentives for management.

#### 2.2.3 Government subsidies

The theory of market failure posits that the state of perfect competition in the market represents an optimal structure for resource allocation. However, market regulation can be prone to blindness and may not fully account for the nuances of economic functions. Consequently, market failure is a phenomenon that exists (36). In the context of market uncertainty, the incentive effect of market competitiveness on innovation is diminished, whereas government subsidies have the potential to reinforce this effect (37). Government subsidies are important in promoting investment and economic growth, restructuring industries, guiding the economy’s direction, maintaining social stability, and creating employment opportunities. By implementing a targeted subsidy policy, the government can effectively disseminate signals pertaining to industrial adjustment and market demand, thereby establishing a clear direction (38). This approach addresses the information asymmetry prevalent between enterprises and investors with regard to R&D and innovation activities, thereby stimulating enterprise innovation activities. The provision of government subsidies to relevant projects will assist enterprises in alleviating the immediate pressure on their investment resources, thereby facilitating the promotion of innovative activities. Consequently, enterprises will enhance their innovative activities in order to increase the likelihood of being awarded further government subsidies (39). In the case of insufficient endogenous financing and limited exogenous financing for enterprises, government subsidies are an important means of injecting capital into enterprises. In the context of economic policy uncertainty, the government provides subsidies to innovative enterprises as a means of conveying the message that innovation is encouraged by the government. This encourages enterprises to enhance their innovation intensity in order to mitigate the risk posed by environmental fluctuations. In light of the aforementioned analysis, the following hypothesis is proposed:

*H4:* The uncertainty of economic policy has an enhancing effect on the investment of firms in innovation, due to an increase in the effectiveness of government subsidy mechanisms.

### 2.3 Manufacturing firms cope with operating costs and business risks

An increase in economic policy uncertainty will result in elevated operating costs and operational risks for enterprises, which will have a detrimental impact on their capacity to enhance innovation intensity and pursue financialization. The extant literature indicates that when economic policy uncertainty increases, enterprises will tend to increase their R&D intensity in order to achieve superior long-term returns and to withstand the impact of the external environment. Meng and Shi (40) posit that economic policy uncertainty will promote corporate innovation, while Xi and Zhang (41) has demonstrated that economic uncertainty has a positive impact on innovation investment through research. However, given the distinctive nature of pharmaceutical manufacturing companies’ innovation activities, which entail a longer cycle and greater risk, the pharmaceutical industry’s profit and revenue growth hinges on the development of proprietary products and innovative drugs. Consequently, enterprises will not curtail their innovation activities, forego necessary innovation costs, or reduce the negative impact of operating costs. Bu et al. (42) identified a discrepancy between the inputs and outputs of innovation. Yan and Hu (43) employed a multiple linear regression model to demonstrate that an increase in economic policy uncertainty prompts enterprises to enhance their innovation inputs. The specificity of pharmaceutical products enables them to reach a distinct consumer group. Consequently, when enterprises intensify their innovation inputs, they can elevate the quality of their pharmaceutical products and accelerate clinical enrolment, thereby enhancing their competitiveness and reducing operational risk. In light of the aforementioned analysis, the following hypotheses are put forth for consideration:

*H5a:* An increase in innovation intensity can serve to mitigate the adverse effects of elevated operational risk faced by firms. However, it is not a panacea for the negative consequences of rising operational costs.

In regard to the financialization of firms, Michael (69) posits that economic policy uncertainty exerts an influence on bank credit, which in turn leads to a narrowing of bank credit, thus increasing the risk of external financing and operational risk for firms. Fatima and Waheed (44) employs a market analysis of Pakistan to demonstrate that economic policy uncertainty precipitates a deterioration in market investment and an increase in the risk of corporate finance. Conversely, policy uncertainty will also serve to increase the degree of information asymmetry between internal and external enterprises, which will produce the ‘lemon effect’ and increase the agency cost within the enterprise (45). Furthermore, it will intensify the discrepancy between the enterprise and the bank’s information, thereby impairing the bank’s capacity to accurately assess the enterprise’s repayment capability (64). This often culminates in delayed or denied lending, which in turn leads to a reduction in operational efficiency and an increase in the enterprise’s operating costs. In order to address the impact of risk and cost, enterprises frequently implement related strategies. Zheng (2021) and others have demonstrated that moderate financialization can stimulate enterprise investment in continuous innovation (65). Xu et al. (46) and others have proposed that financialization reduces the level of enterprise risk-taking, but it also results in the withdrawal of industrial capital from the real business, which has an adverse impact on competition for the enterprise’s products and sustainable development. The process of financialization enables enterprises to increase their internal financing, reduce their dependence on external financing, and obtain excess returns. It also allows them to reduce their operating costs and increase their financial investment costs. However, there is a risk that if financialization is overused in the long term, it could result in a shift from the real economy to the virtual economy. An increase is to be expected. The following hypothesis is put forth for consideration:

*H5b:* Financialization may continue to accommodate the risk implications of economic policy uncertainty; however, the response effect will be diminished with the escalation of financialization risk. Due to the lagged nature of the investment, financialization serves to mitigate the impact of cost shocks in subsequent periods; however, it is not a panacea for addressing cost shocks in the current period.

## 3 Research design

### 3.1 Model setting and sample selection

In order to identify the impact of economic policy uncertainty on enterprise innovation, this paper uses the fixed effect model to construct the following econometric model:


(1) $$RD_{i,t}=\beta_{0}+\beta_{1}EPU_{t}+\mathrm{\delta Contra}l_{i,t}+\mu_{i}+\varepsilon_{i,t}$$

The subscripts *i* and *t* in its Equation 1 denote firms and years, respectively, with economic policy uncertainty as the explanatory variable and firm innovation as the explanatory variable, Control denotes the set of control variables, $\mu_{i}$ is the firms’ individual fixed effect, and $\varepsilon_{i,t}$ is the random perturbation term. Referring to the approach of Wang and Tian (47). In this paper, we refrain from controlling for time-fixed effects due to the fact that the economic policy uncertainty index is a country-level time series, which would inevitably lead to the issue of multicollinearity if we were to control for time fixed effects. The regression coefficient $\beta_{1}$ , as shown in the above regression equation, is of particular interest. A positive $\beta_{1}$ indicates that an increase in economic policy uncertainty encourages firms to pursue digital transformation, whereas a negative β_1 implies that it hinders such efforts.

In this paper, A-share listed companies in the pharmaceutical manufacturing industry from 2015 to 2022 are selected as the initial research sample to explore the relationship between economic policy uncertainty and corporate innovation and the relationship between financialization. In order to ensure the data validity, the sample is processed as follows: firstly, financial companies are excluded; secondly, samples with ST and period delisting are excluded; thirdly, samples of companies with missing key variables for three consecutive years are excluded, and finally, 1,128 observation samples are obtained. The raw data are all from the Cathay Pacific database (CSMAR) and the China Research Data Service Platform (CNRDS), and the relevant financial data are from the RESSET database.

### 3.2 Mechanism analysis model setting

In order to determine the impact of economic policy uncertainty on enterprise innovation, drawing on the idea of Jiang (60), this paper constructs the following model:


(2) $$FIN_{i,t}/PAY_{i,t}=\alpha_{0}+\alpha_{1}EPU_{t}+\mathrm{\delta Contra}l_{i,t}+\mu_{i}+\varepsilon_{i,t}$$

(3) $$\begin{array}{l} RD_{i,t}=\\ \omega_{0}+\omega_{1}EPU_{t}+\omega_{2}FIN_{i,t}/PAY_{i,t}+\mathrm{\delta Contra}l_{i,t}+\mu_{i}+\varepsilon_{i,t} \end{array}$$

Where (Equation 2) FIN, PAY denotes corporate financialization and executive incentives, Control denotes the set of control variables, $\mu_{i}$ is the firm’s individual fixed effect, and $\varepsilon_{i,t}$ is the random perturbation term. The model allows for the verification of the impact of economic policy uncertainty on firms’ innovation intensity. It is essential to ascertain whether the $\omega_{1}$ and $\alpha_{1}$ coefficients are statistically significant in order to determine whether the effect is positive or negative.

### 3.3 Definition of variables

#### 3.3.1 Explained variables

Firm Innovation (RD). Academics divide the indicators for measuring enterprise innovation into R&D input and R&D output. The timeframe from R&D input to patent approval is very long. In contrast, the enterprise innovation studied in this paper is more interested in the enterprise’s willingness to innovate in the case of economic policy uncertainty. Hence, this paper draws on the practice of Sun and Chen (49) and adopts the ratio of the enterprise’s R&D investment in operating revenues as an indicator of enterprise innovation.

#### 3.3.2 Explanatory variables

Economic policy uncertainty (EPU). The Baker et al. (68) team counted, developed, and published an index of China’s economic policy uncertainty by searching for keywords on the South China Morning Post reports. The index is widely used in existing research, has been extensively tested and applied, and has been proven to have a certain degree of validity and stability. Since the index is a monthly index, this paper obtains the annual index by taking the arithmetic mean after summing the monthly data of each year and dividing the index by 100 to ensure the consistency of the data.

#### 3.3.3 Control variables

The innovation of an enterprise is affected by the enterprise’s own resources. In this paper, we adopt the following control variables: firm size (SIZE), defined as the natural logarithmic value of the enterprise’s total assets; enterprise liability ratio (LEV), defined as the ratio of the enterprise’s current year liabilities to total assets, which is used to measure the enterprise’s level of financial risk. The proportion of the largest shareholder’s shareholdings (TOP), defined as the ratio of the number of shares held by the largest shareholder to the total number of shares, the willingness of the largest shareholder exerts a significant influence on the implementation of the enterprise’s innovation project and the importance of the project. The Tangible Assets Ratio (YX), defined as the total amount of tangible assets to the ratio of the total assets, provides insight into the structure of the enterprise. The Management Expense Ratio (GL) is defined as the ratio of management expenses to total assets and is used to reflect the management style of the company. The Cash Flow Ratio (FCF) is defined as the ratio of cash flow to total assets and shows the capital status of the company. Tobin’s Q (TQ) is defined as the ratio of the company’s market capitalization to total assets and is used to reflect the market value of the company. The growth rate of operating income (YY) provides insight into the growth trajectory of the firm. Table 1 delineates the variable definitions.

**Table 1:** Definition of variables.

Variable name	Variable symbol	Description of variable
Firm innovation	RD	Firm’s R&D investment as a percentage of operating income
Economic policy uncertainty	EPU	Statistics and formulation of SCMP reports by searching keywords
Firm size	SIZE	Natural logarithmic value of firm’s total assets
Corporate debt ratio	LEV	Ratio of current year’s liabilities to total assets of a company
TOP ratio of shares held by the largest shareholder	TOP	Ratio of the number of shares held by the largest shareholder to the total number of shares
Ratio of tangible assets	YX	Ratio of total tangible assets to total assets
Administrative expense ratio	GL	Administrative expense to total assets of the enterprise
Cash flow ratio	FCF	Ratio of cash flow to total assets
Operating income growth rate	YY	(Operating income current year current single quarter amount − Operating income previous single quarter amount)/Operating income previous single quarter amount
Tobin’s Q	TQ	Ratio of firm’s market capitalisation to total assets
Financialization	FIN	Ratio of financial assets to total corporate assets
Government subsidies	BT	Ratio of government subsidies to total assets or operating income
Executive incentives	PAY	The natural logarithm of the total compensation of the top three executives in the firm’s payroll
Innovation output	PAT	The logarithm of the number of patent applications filed by the company in the year +1.
Cost	COST	Operating Costs to Operating Revenue Ratio
Operating risk	RISK	Three-year rolling standard deviation of total net asset margins

## 4 Analysis of empirical results

### 4.1 Descriptive statistics

The descriptive statistics of the variables are shown in Table 2. The mean value of the enterprise innovation R&D investment is 6.18%, and the standard deviation is 7.87%, which shows that the overall R&D investment among the sample enterprises is not high. The maximum and minimum values of the sample are a significant difference in R&D investment. From 2015 through 2022, the mean value of China’s economic policy uncertainty is 5.17, and the standard deviation is 0.10, which shows that the degree of China’s economic policy changes is noticeable. Meanwhile, the mean value of financialization is 7.60%, and the standard deviation is 10.41%. The overall degree of financialization is low, and the sample’s maximum and minimum values can show a significant difference in financialization development between the samples.

**Table 2:** Results of descriptive statistics.

Variable	Sample size	Mean	Standard deviation	Minimum	Maximum
RD	1,128	0.0618	0.0787	0.0001	1.6700
EPU	1,128	5.1700	2.0360	1.8120	7.9190
SIZE	1,128	21.5437	1.1162	18.5786	24.9796
LEV	1,128	0.3217	0.1708	0.0143	0.8858
TOP	1,128	32.1247	13.0934	4.1800	69.1600
YX	1,128	0.8994	0.0977	0.4191	1.0000
GL	1,128	0.1013	0.0751	0.0054	1.3601
FCF	1,128	0.0694	0.6151	−0.1487	0.4706
YY	1,128	0.1831	1.0947	−1.6723	32.9643
TQ	1,128	2.5986	1.9424	0.7152	22.5724
FIN	1,128	0.0761	0.1041	0	0.7556
BT	1,128	0.1253	0.1564	0	0.1919
PAY	1,128	14.8154	0.7181	12.5707	17.6935
PAT	1,128	2.5872	1.2684	0.0000	6.2672
COST	1,128	0.4875	0.4277	0.0526	7.1063
RISK	791	0.5145	0.0510	0	0.5130

### 4.2 Correlation analysis

Table 3 demonstrates the Spearman correlation coefficients between the variables. The VIF values are all less than 5, indicating no severe multicollinearity problem in the data.

**Table 3:** Results of variable correlation coefficient test.

	RD	LNPU	FIN	LEV	FCF	TQ	TOP	SIZE	GL	YX	YY
RD	1										
LNPU	0.088***	1									
FIN	0.067**	0.161***	1								
LEV	−0.069**	0.063**	−0.189***	1							
FCF	−0.079***	0.041	−0.004	−0.302***	1						
TQ	0.118***	−0.153***	0.003	−0.282***	0.233***	1					
TOP	−0.076**	−0.083***	−0.051*	−0.143***	0.207***	0.108***	1				
SIZE	−0.134***	0.149***	−0.036	0.236***	0.180***	−0.149***	0.158***	1			
GL	0.216***	−0.162***	0.028	0.085***	0.214***	0.042	−0.107***	−0.437***	1		
YX	0.022	0.105***	0.070**	0.007	0.047	0.098***	0.04	0.077***	−0.074**	1	
YY	−0.022	−0.059**	−0.048	0.024	0.01	−0.049*	−0.044	−0.022	0.043	−0.007	1

*p < 0.1, **p < 0.05, ***p < 0.01.

### 4.3 Benchmark regression analysis

Table 4 reports the results of the benchmark regression of economic policy uncertainty on innovation in pharmaceutical manufacturing firms. Columns (1) and (2) present the results of the benchmark regression of economic policy uncertainty on innovation of pharmaceutical manufacturing firms. Column (1) presents the estimation results with only economic policy uncertainty included when the regression coefficient of economic policy uncertainty is significantly positive. The results in column (2) show that the economic policy uncertainty regression coefficient is still significantly positive after adding control variables. This paper chooses column (2) as the baseline regression estimation results. The results show that the regression coefficient of economic policy uncertainty is 0.0042, which is significant at a 5% statistical level. That is, economic policy uncertainty positively impacts the innovation of pharmaceutical manufacturing enterprises, which indicates that the rise of economic policy uncertainty presents the promotion of innovation investment in the pharmaceutical manufacturing industry. This result has the same significant economic result is also economically significant. When the economic policy uncertainty faced by firms increases by one standard deviation (2.03), firms are expected to increase the level of innovation investment by 1.16% (0.0057 × 2.03), which is 18.77% of the average innovation investment of pharmaceutical firms (6.18%). This result suggests that when facing the challenges posed by economic policy uncertainty, firms are more inclined to seize the opportunities presented by innovation, further improve the market competitiveness of pharmaceutical firms by increasing innovation investment, and obtain more resources to be lower than the negative impact of economic policy uncertainty. The hypothesis H1 of this paper is verified.

**Table 4:** Results of the baseline regression of economic policy uncertainty and innovation intensity.

	(1)	(2)
	RD	RD
LNPU	0.0034***	0.0057***
	(0.0011)	(0.0017)
LEV		−0.0385
		(0.0323)
FCF		−0.182**
		(0.0763)
TQ		−0.0001
		(0.0014)
TOP		0.0006
		(0.0006)
SIZE		−0.0071
		(0.0124)
GL		0.155*
		(0.0884)
YX		−0.0156
		(0.0401)
YY		−0.0007
		(0.0009)
Constant	0.0442***	0.191
	(0.0057)	(0.258)
N	1,128	1,128
*R* ^2^	0.013	0.063
Adj. *R*^2^	0.013	0.055

*p < 0.1, **p < 0.05, ***p < 0.01.

### 4.4 Robustness test

In order to improve the reliability of the estimation results of the research in this paper, the stability of the regression results is tested by using the replacement of the explanatory variables, the method of shrinking the tails and the replacement of the regression model. The results of the robustness tests are presented in Table 5.

**Table 5:** Robustness test results.

	Replacement of the explanatory variables	Tailoring	Substitution of explanatory variables	Replacement regression method
LNPU coefficient	0.0019^***^	0.0044^***^	0.0124^***^	0.0056^***^
Control variable	Yes	Yes	Yes	Yes
Fixed effect	Yes	Yes	Yes	No
Sample size	1,128	843	1,128	1,128
*R* ^2^	0.135	0.146	0.065	0.093
Adj. *R*^2^	0.128	0.137	0.057	0.086

*p < 0.1, **p < 0.05, ***p < 0.01.

#### 4.4.1 Replacement of explanatory variables

In order to avoid the influence of the calculation of innovation input on the regression results, this paper, following the practice of Li et al. (50), Gao et al. (23), redefines the innovation input of the enterprise as the ratio of R&D investment to the total assets of the enterprise in the current period and redefines the innovation input as an explanatory variable to retest the H1 hypothesis, and the results are shown in Table 5. As can be seen from the figure, the results are significantly positive with the benchmark regression, and economic policy uncertainty helps to promote firms’ innovation investment.

#### 4.4.2 Tailoring

In order to eliminate the impact of extreme values, this paper uses the shrink-tailed processing method to detect the robustness of the benchmark results, this paper on continuous variables are at the 1% level Winsorize shrink-tailed processing, the results are shown in Table 5, the regression results and the benchmark regression results are all significantly positive, that is, economic policy uncertainty has a facilitating effect on pharmaceutical firms’ innovation.

#### 4.4.3 Replacement of regression methods

The base regression in this paper chooses the fixed effect model, so the OLS model is used to conduct the robustness test, and the results are shown in Table 5. The results from the table are the same as the results of the base regression, the coefficient of economic policy uncertainty is significantly positive, that is, in the face of increasing economic policy uncertainty, pharmaceutical manufacturing enterprises choose to increase investment in innovation to improve their market competitiveness to withstand the challenges brought by external environmental shocks.

#### 4.4.4 Replacing explanatory variables

Davis et al. constructed a new set of economic policy uncertainty indices for China by keyword searching information from People’s Daily and Guangming Daily. In order to avoid the influence of the calculation method of the economic policy uncertainty index on the results of the benchmark regression, this paper replaces the calculation method of the economic policy uncertainty index with that of Davis and uses the index as the core explanatory variable for the regression, and the regression results are shown in Table 5. Table 5 shows that the estimated coefficients of the explanatory variables are still significantly positive, indicating that the results of the benchmark regression are robust.

### 4.5 Endogeneity test

The estimation results of this paper may have endogeneity problems mainly caused by the following aspects: firstly, it is difficult to control all the factors affecting the innovation of pharmaceutical manufacturing enterprises in this paper, and omitted variables cause endogeneity problems; secondly, the estimation results of this paper may have reverse causality problems. For the problem of omitted variables in the regression model, this paper employs a fixed-effects model in the benchmark regression, which mitigates the impact of omitted variables on the estimation results to a certain extent.

#### 4.5.1 Instrumental variable method

Following Peng et al. (51), this paper constructs instrumental variables by selecting the economic policy uncertainty of China’s major trading countries. This paper selects the economic policy uncertainty data of seven countries, including Germany, France, Italy, Japan, South Korea, the United States, and the United Kingdom, for the period 2015–2022. The economic policy uncertainty of the above countries is weighted according to the weight of the annual import and export volume with China, and the economic policy uncertainty indices obtained after weighting are aggregated to derive an aggregated economic policy uncertainty variable as an instrumental variable, and a two-stage regression is conducted. The logic of selecting these variables is that the economic policy uncertainty of these countries may affect China’s economic policy uncertainty through the trade channel, and studies such as Li et al. (50) and Gu et al. (52) have proved that the economic policy uncertainty of other countries is highly unlikely to directly affect the business strategies of firms. Therefore, this instrumental variable is exogenous and relevant and can be used as an instrumental variable. Based on this, this paper conducts 2SLS regression with the weighted economic policy uncertainty of major trading countries as the instrumental variable, where the results of the first stage regression are shown in column (1) of Table 6, and the regression coefficients of the instrumental variable are significantly positive, and the results of the second stage regression are shown in column (2), the regression coefficients of the economic policy uncertainty index are significantly positive at the 1% level, and the F-statistic value of the instrumental variable is greater than the critical value of the Stock-Yogo weak identification test at the 10% level, indicating that the instrumental variable is not a weak instrumental variable.

**Table 6:** Instrumental variables approach.

Variables	(1)	(2)
	LNPU	RD
LNPU		0.0056***
IV	3.898***	
Control variable	Yes	Yes
Fixed Effects	Yes	Yes
Sample size	1,128	1,128
*R*^2^ value		0.0860
F-statistic		20359.03

*p < 0.1, **p < 0.05, ***p < 0.01.

#### 4.5.2 One-period lagged test of independent variables

In order to avoid reverse causality, this paper adopts a one-period lag treatment for the economic policy uncertainty index to circumvent the endogeneity problem and conducts regression analysis using the fixed effect model, as shown in Table 7, the economic policy uncertainty index is still positively correlated with the innovation intensity of the enterprise, and is significant at the 5% level.

**Table 7:** Lagged one-period regression of independent variables to test the endogeneity problem.

	(1)	(2)
	RD	RD
L.LNPU	0.0029**	0.0046***
	(0.0012)	(0.0015)
LEV		−0.0275
		(0.0389)
FCF		−0.168**
		(0.0721)
TQ		−0.0009
		(0.0018)
TOP1		0.0007
		(0.0007)
SIZE1		−0.0074
		(0.0144)
GL		0.158*
		(0.0944)
YX		−0.0418
		(0.0478)
YY		−0.0010
		(0.001)
Constant	0.05***	0.223
	(0.0058)	(0.3)
N	987	987
*R* ^2^	0.009	0.055
Adj. *R*^2^	0.008	0.047

*p < 0.1, **p < 0.05, ***p < 0.01.

### 4.6 Heterogeneity analysis

#### 4.6.1 Management heterogeneity within firms

This paper conducts sample regressions by dividing the sample into the presence of two jobs, the absence of two jobs, the presence of overseas background, and the absence of overseas background based on the presence of two jobs for the management of the company and the presence of overseas background for the senior management. The results are shown in Table 8, where columns (1) and (2) are analyzed for the presence or absence of overseas background of management, and columns (3) and (4) are analyzed for the presence or absence of two-job integration of management. Firms with overseas backgrounds and dual jobs among executives are more likely to choose to increase the innovation intensity of their firms in the face of economic policy uncertainty than firms with no overseas backgrounds or dual jobs among executives. On the one hand, firms with two jobs tend to respond and adjust faster to external shocks or related policy changes. In contrast, firms without two jobs need to hold meetings to discuss shocks caused by the external environment, and their decision-making paths are longer, preventing them from making immediate decisions and delaying the time for the firm to make the optimal outcome. On the other hand, the fact that executives have overseas backgrounds indirectly verifies that executives have diversified educational backgrounds, are experienced in dealing with policy shifts, and have a wealth of theoretical knowledge in reserve so that they can grasp the general direction of the company, jump out of the existing thinking, and provide solutions to address external shocks. Therefore, a firm’s management with a combination of both positions and an overseas background is more likely to significantly increase the firm’s innovation intensity to cope with risks when economic policy uncertainty rises.

**Table 8:** Impact of management heterogeneity within firms.

	(1)	(2)	(3)	(4)
	No overseas background	Overseas background	Non-dual employment	Dual employment
LNPU	0.0011	0.0051*	0.004	0.0039***
	(0.0007)	(0.0028)	(0.0029)	(0.0014)
LEV	−0.0432**	−0.0563	−0.0212	−0.0404
	(0.0165)	(0.0478)	(0.0477)	(0.0254)
FCF	−0.0273	−0.260**	−0.168	−0.159***
	(0.0251)	(0.112)	(0.124)	(0.0564)
TQ	−0.0033***	0.0001	0.0009	0.0008
	(0.001)	(0.0013)	(0.0022)	(0.0027)
TOP1	0.0002	0.0002	−0.0001	0.00162**
	(0.0002)	(0.0006)	(0.0005)	(0.0007)
SIZE	−0.0034	0.0238	0.0148	0.0067
	(0.0076)	(0.0159)	(0.0152)	(0.0092)
GL	0.0715	0.375***	0.409***	0.141**
	(0.0504)	(0.0686)	(0.116)	(0.0645)
HB	0.0011*	−0.0008	−0.003*	0.0008
	(0.0006)	(0.0015)	(0.0017)	(0.0014)
YX	0.0356	−0.0185	−0.0204	0.0763
	(0.0286)	(0.0598)	(0.0499)	(0.0480)
Constant	0.0838	−0.473	−0.265	−0.221
	(0.183)	(0.330)	(0.321)	(0.216)
N	378	750	801	327
*R* ^2^	0.201	0.093	0.059	0.199
Adj. *R*^2^	0.182	0.082	0.048	0.176

*p < 0.1, **p < 0.05, ***p < 0.01.

#### 4.6.2 Enterprise ownership heterogeneity

In this paper, the sub-sample of SOEs and the sub-sample of non-SOEs are divided according to the type of ownership, and the sub-samples are regressed. Table 9 shows that non-state-owned enterprises tend to significantly increase their innovation intensity in the face of economic policy uncertainty compared to state-owned enterprises. The possible explanation for this is that due to the large size of SOEs and the complexity of their internal systems, SOEs face insignificant external risks and do not need to invest in innovation to capture the value of growth due to the long reaction time and dependence on national policies to cope with external shocks. Demand for reform and innovation is not strong, which has a negative impact on improving innovation intensity. In addition, SOEs are relatively less affected by the external policy environment and market competition and tend not to suddenly increase their investment in innovation, but continue to invest according to their previous plans. In contrast, in the face of external shocks, non-state enterprises tend to be more willing to optimize the internal environment and resist shocks and will increase the intensity of innovation investment in ongoing research projects in order to increase their market competitiveness and better cope with uncertainty.

**Table 9:** Analysis of ownership heterogeneity.

	(1)	(2)
	Non-SOEs	SOEs
LNPU	0.0055^**^	0.0003
	(0.0025)	(0.0007)
LEV	−0.0564	−0.0230^*^
	(0.0403)	(0.0127)
FCF	−0.254^**^	−0.0220
	(0.121)	(0.0165)
TQ	0.001	−0.0016^**^
	(0.0016)	(0.0007)
TOP1	0.0006	0.0006^**^
	(0.0007)	(0.0003)
SIZE	0.0171	0.0171^***^
	(0.0133)	(0.0056)
GL	0.327^***^	0.0667^*^
	(0.0623)	(0.0339)
HB	−0.0018	0.0004
	(0.0016)	(0.0004)
YX	0.0054	−0.0153
	(0.0405)	(0.0290)
Constant	−0.346	−0.355^**^
	(0.284)	(0.139)
*N*	840	288
*R* ^2^	0.087	0.198
Adj. *R*^2^	0.077	0.173

*p < 0.1, **p < 0.05, ***p < 0.01.

## 5 Further analysis

### 5.1 Internal mechanisms of economic policy uncertainty affecting innovation in pharmaceutical manufacturing firms

Based on the analysis of the theory, increasing economic policy uncertainty strengthens the incentives for pharmaceutical manufacturing firms to innovate, and this is because economic policy uncertainty induces firms to undertake financialization shifts. Therefore, this paper first tests whether economic policy uncertainty induces financialization shifts within firms. Based on the idea of Jiang (48), the following model is constructed:


(4) $$FIN_{i,t}/PAY_{i,t}=\alpha_{0}+\alpha_{1}EPU_{t}+\mathrm{\delta Contra}l_{i,t}+\mu_{i}+\varepsilon_{i,t}$$

According to the above model, observing whether $\alpha_{1}$ in the equation is significant or not, it can be determined whether internal mechanisms are affected by economic policy uncertainty. Secondly, the above mechanism variables are included in the benchmark regression, which in turn tests whether the internal financialization of firms under economic policy uncertainty promotes firms to increase innovation intensity. The relevant econometric model is as follows:


(5) $$RD_{i,t}=\omega_{0}+\omega_{1}EPU_{t}+\omega_{2}FIN_{i,t}+\mathrm{\delta Contra}l_{i,t}+\mu_{i}+\varepsilon_{i,t}$$

(6) $$RD_{i,t}=\gamma_{0}+\gamma_{1}EPU_{t}+\gamma_{2}PAY_{i,t}+\mathrm{\delta Contra}l_{i,t}+\mu_{i}+\varepsilon_{i,t}$$

Observe whether the coefficients of the economic policy uncertainty index in Equations 5 and 6 are significant. On the degree of financialization (FIN). Based on Zhai and Dai (53) and others, it is measured by the ratio of financial assets to total assets of the company. Financial assets include trading financial assets, available-for-sale financial assets, derivative financial assets, long-term equity investments, held-to-maturity investments, and investment real estate, and the larger the indicator, the higher the business risk of the firm; for executive compensation, drawing on Zhu et al. (54), the natural logarithm of the total compensation of the firm’s top three executives in terms of compensation is used to measure it, and the higher the indicator, the higher the level of incentive compensation. The control variables in Equations 2–5 are consistent with the benchmark regression.

Columns (1) and (2) of Table 10 present the estimation results of economic policy uncertainty on the degree of financialization of firms and executive compensation incentives. Based on the results in column (1), we can see that the coefficient of the effect of economic policy uncertainty on the degree of firm financialization is significantly positive, with a coefficient of 0.0067, i.e., an increase in economic policy uncertainty increases the degree of firm financialization. From column (2), the regression coefficient of economic policy uncertainty on firms’ executive compensation incentives is 0.0703 and significant at the 1% level, i.e., an increase in economic policy uncertainty causes firms to increase executive compensation. It indicates that firms increase internal financialization and executive compensation when faced with economic policy uncertainty. Columns (3)(4) of Table 10 include financialization and executive compensation in the baseline regression, and from the results in column (3), the regression coefficient of financialization is 0.0555, which is significant and positive at the 5% level. The coefficient of economic policy uncertainty on firm innovation is also significant and positive, with a coefficient of 0.0053, which is lower than that in the baseline regression. It indicates that economic policy uncertainty leads firms to improve their innovation intensity by increasing their financialization. From the results in column (4), it can be seen that the regression coefficient of executive compensation is significantly positive when it is included in the base regression, and the coefficient of economic policy uncertainty on firms’ innovation is 0.0042, which is significantly positive at the 5% level and lower than that of the base regression. It indicates that economic policy uncertainty will lead firms to improve their innovation strength by increasing the compensation of managers within the firm.

**Table 10:** Internal mechanism of economic policy uncertainty on innovation in pharmaceutical manufacturing enterprises.

	(1)	(2)	(3)	(4)
	FIN	PAY	RD	RD
LNPU	0.0067^***^	0.0703^***^	0.0053^***^	0.0042^**^
	(0.0019)	(0.001)	(0.0017)	(0.0019)
LEV	−0.0292	−0.337^*^	−0.0369	−0.0312
	(0.0354)	(0.172)	(0.0319)	(0.0303)
FCF	0.0027	0.146	−0.182^**^	−0.185^**^
	(0.0626)	(0.277)	(0.0761)	(0.0752)
TQ	−0.0002	−0.0063	−0.0001	0.0001
	(0.0038)	(0.0081)	(0.0014)	(0.0015)
TOP1	−0.0013	−0.0017	0.0007	0.0006
	(0.0009)	(0.0047)	(0.0006)	(0.0005)
SIZE	0.0056	0.357^***^	−0.0075	−0.0149
	(0.0128)	(0.0739)	(0.0122)	(0.0113)
GL	0.0612	1.078^***^	0.152^*^	0.132
	(0.0799)	(0.287)	(0.0856)	(0.0896)
YX	0.160^*^	0.533^**^	−0.0244	−0.0271
	(0.0941)	(0.253)	(0.0411)	(0.0439)
YY	−0.003^***^	−0.0220^*^	−0.0005	−0.0002
	(0.0008)	(0.0111)	(0.001)	(0.0011)
FIN			0.0555^**^	
			(0.0256)	
PAY				0.0216^**^
				(0.0092)
Constant	−0.177	6.350^***^	0.201	0.0533
	(0.303)	(1.615)	(0.253)	(0.274)
*N*	1,128	1,128	1,128	1,128
*R* ^2^	0.099	0.332	0.066	0.076
Adj. *R*^2^	0.091	0.327	0.058	0.068

*p < 0.1, **p < 0.05, ***p < 0.01.

### 5.2 External mechanisms of economic policy uncertainty affecting innovation in pharmaceutical manufacturing firms

According to the theoretical analysis, increasing economic policy uncertainty increases the incentives for pharmaceutical manufacturing firms to innovate because economic policy uncertainty induces the government to intervene and subsidize firms. Therefore, this paper tests whether economic policy uncertainty leads the government to provide subsidies. Specifically, the following model is constructed:


(7) $$\mathrm{Go}v_{i,t}=\rho_{0}+\rho_{1}EPU_{t}+\mathrm{\delta Contra}l_{i,t}+\mu_{i}+\varepsilon_{i,t}$$

It is shown according to Equation 7, observing whether $\rho_{1}$ in the equation is significant or not, it can be determined whether internal mechanisms are affected by economic policy uncertainty. Second, we include the above mechanism variables in the benchmark regression and then test whether government subsidies induce firms to increase innovation intensity under economic policy uncertainty. The econometric model is as follows:


(8) $$RD_{i,t}=\theta_{0}+\theta_{1}EPU_{t}+\theta_{2}\mathrm{Go}v_{i,t}+\mathrm{\delta Contra}l_{i,t}+\mu_{i}+\varepsilon_{i,t}$$

Observe whether the coefficients of the economic policy uncertainty index in Equation 8 are significant. Following the practice of Xie and Caij (55), the ratio of government subsidies to total assets or operating income is used as a measure, and the larger the ratio, the more government subsidies are invested.

Column (1) of Table 11 presents the estimation results of economic policy uncertainty on government subsidies. The results show that the regression coefficient of economic policy uncertainty is positive and significant at the 1% level, indicating that an increase in economic policy uncertainty leads to an increase in government subsidies. Column (2) includes government subsidies in the benchmark regression. The results show that the regression coefficient of government subsidies on gold is significantly positive when government subsidies are included in the benchmark regression. The coefficient of economic policy uncertainty on firm innovation is also significantly positive, indicating that economic policy uncertainty induces firms to improve their own innovation intensity by increasing government subsidies.

**Table 11:** External mechanisms of economic policy uncertainty affecting innovation in pharmaceutical manufacturing firms.

	(1)	(2)
	BT	RD
LNPU	0.001^***^	0.0053^***^
	(0.0002)	(0.0017)
LEV	0.0074	−0.0416
	(0.0057)	(0.0312)
FCF	0.0021	−0.183^**^
	(0.0095)	(0.0761)
TQ	−0.0004	0.0001
	(0.0005)	(0.0013)
TOP	−0.0001	0.0006
	(0.0001)	(0.0006)
SIZE	−0.0034^**^	−0.0057
	(0.0015)	(0.0119)
GL	0.0128	0.150^*^
	(0.0193)	(0.0837)
YX	−0.0073	−0.0125
	(0.0097)	(0.0401)
YY	−0.0003	−0.0005
	(0.0002)	(0.0009)
BT		0.424^*^
		(0.231)
Constant	0.0863^**^	0.154
	(0.0356)	(0.249)
*N*	1,128	1,128
*R* ^2^	0.045	0.069
Adj. *R*^2^	0.037	0.061

*p < 0.1, **p < 0.05, ***p < 0.01.

### 5.3 Coping with operating cost shocks from economic policy uncertainty

Based on the analysis of internal mechanisms, this paper argues that firms increase the intensity of innovation investment by alleviating the financial pressure through internal financialization. Based on the theoretical analysis in the previous section, this paper argues that firms’ innovation can cope with the negative shock to operating costs caused by increasing economic policy uncertainty. To test the above hypothesis from the perspective of whether firms can cope with the shocks caused by economic policy uncertainty by enhancing their advantages, the results are shown in Table 12.

**Table 12:** An analysis of the impact of operating cost shocks resulting from economic policy uncertainty on business strategies.

Variant	(1)	(2)	(3)	(4)
	D.CB	LD.CB	D.CB	LD.CB
RD	−12.45	−10.53		
	(510.97)	(58.07)		
FIN			−9.26	−7.83***
			(380.22)	(43.2)
Control variable	Yes	Yes	Yes	Yes
Fixed effects	Yes	Yes	Yes	Yes
Sample size	987	846	987	846
*R* ^2^	0.118	0.104	0.118	0.104
Adj. *R*^2^	0.106	0.091	0.106	0.091

*p < 0.1, **p < 0.05, ***p < 0.01.

Based on the research ideas of Zhu et al. (56) and others, this paper adopts a two-stage approach to analyze the economic effects of firms’ innovation intensity to support the hypothesis. In the first step, the baseline regression is transformed into a differential form. Then, the change in firms’ innovation intensity caused by the change in economic policy uncertainty is estimated:


(9) $$\Delta RD/FIN_{i,t}=\omega_{0}+\Delta EPU_{i,t}+\mathrm{\delta Contra}l_{i,t}+\mu_{i}+\varepsilon_{i,t}$$

In Equation 9, the fitted value of $\Delta RD_{i,t}$ is the real impact of economic policy uncertainty on firms’ innovation intensity. In the second step, the following econometric model is constructed to estimate the impact of the change in firms’ innovation intensity due to the increase in economic policy uncertainty on firms’ operating costs, and then to test the economic consequences of the change in economic policy uncertainty.


(10) $$\frac{\mathrm{\Delta cos}t_{i,t}}{\mathrm{\Delta cos}t_{i,t+1}}=\pi_{0}+\Delta \bar{RD_{i,t}/FIN_{i,t}}+\mathrm{\delta Contra}l_{i,t}+\mu_{i}+\mu_{t}+\varepsilon_{i,t}$$

In Equation 10, $\mathrm{\Delta cos}t_{i,t}$ represents the difference between $\cos t_{i,t}$ and $\cos t_{i,t+1}$ . The enterprise operating cost (cost) is calculated using the method proposed by Ye et al. (57) employs the operating cost ratio of operating income as a measure of enterprise operating risk (risk), which draws on the practice of Xin et al. (58) and utilizes the three-year rolling standard deviation of the total assets net interest rate.

The estimates of the relationship between firms’ increased innovation intensity and their operating costs in the current and future periods, respectively, are presented in columns (1) and (2) of Table 12. The effect of innovation inputs on current and future operating costs is found to be insignificant, with coefficients of −12.45 and −10.53, respectively. Columns (3) and (4) present the results of the estimation of the financialization indicator of the firms in relation to the change in operating costs in the current and future periods, respectively. The impact of financialization transformation within the firm on current period operating costs is inconsequential, with a coefficient of −12.45. However, the effect on future period operating costs is markedly negative, with a coefficient of −10.53. This suggests that increased investment in innovation is unable to offset negative shocks to operating costs caused by economic policy uncertainty. Financialization is unable to cope with the current cost shocks resulting from economic policy uncertainty. However, it is capable of coping with shocks to future operating costs. The reason may be that, although economic policy uncertainty has a negative impact on operating costs, the specific characteristics of innovation within the pharmaceutical industry, including the lengthy research and development cycle of new products, the stability of capital investment, and the increasing operating costs, prevent innovation investment from solving the problem of rising operating costs. Furthermore, financial transformation through the continuous configuration of its own financial resources serves to alleviate the phenomenon of financial mismatch, thereby reducing the cost impact of economic policy uncertainty.

The test results substantiate the hypothesis that pharmaceutical manufacturing enterprises are unable to mitigate the adverse effects of rising operational costs resulting from economic policy uncertainty through enhanced innovation intensity. Conversely, financialization has the potential to serve as a resilience mechanism against the anticipated negative impact of rising operational costs in the future.

### 5.4 Coping with operational risk from economic uncertainty

In light of the aforementioned analyses, it is postulated that firms are capable of adapting to adverse effects resulting from an increase in economic policy uncertainty. To substantiate this hypothesis, the same methodology is employed to conduct further tests, the outcomes of which are presented in Table 13.

**Table 13:** An analysis of the effects of coping with business risk shocks from economic policy uncertainty.

Variant	(1)	(2)	(3)	(4)
	D.risk	LD.risk	D.risk	LD.risk
rd_hat	−1053.4^***^	−79.6^***^		
	(222.07)	(28.34)		
FIN1_hat			−783.9^***^	−59.2^***^
			(164.30)	(21.08)
Control variable	Yes	Yes	Yes	Yes
Fixed effects	Yes	Yes	Yes	Yes
Sample size	705	564	705	564
*R* ^2^	0.024	0.031	0.024	0.031
Adj. *R*^2^	0.009	0.013	0.009	0.013

*p < 0.1, **p < 0.05, ***p < 0.01.

Columns (1) and (2) of Table 13 present the estimated results of the business risk associated with firms’ increased innovation intensity in the current period and the future period, respectively. The results demonstrate that there is a significantly negative correlation between firms’ innovation intensity and business risk in both the current and future periods. The coefficients for the current period are notably lower than those for the future period, with values of −1053.4 and −79.6, respectively. This suggests that investment in innovation can serve to mitigate current and future business risks for enterprises, although the impact may diminish over time. This may be attributed to the fact that enterprises gain a competitive advantage by increasing their innovation intensity. Pharmaceutical enterprises enhance product competitiveness by increasing innovation investment and innovation pipeline research, which also strengthens enterprise confidence. This, in turn, prompts enterprises to adjust internal management and organizational structure and motivates management to enhance management work enthusiasm. The lengthy innovation cycle of the pharmaceutical industry, coupled with the fact that innovation funds are not utilized once, but rather at various stages of the project, and that the subjects of the drugs in question must be evaluated over an extended period of time, means that the output of innovation does not serve to mitigate the risk faced by the enterprise in a timely manner. Consequently, the enterprise is still required to rely on the original product in order to generate profits for a period of time. Furthermore, augmented innovation investment disrupts the established market competition pattern. Consequently, enterprises must contend with heightened competitive risk, which in turn elevates the enterprise’s future business risk. In the long term, the impact of innovation investment in mitigating risk will diminish.

Table 13, columns (3) and (4), present the estimation results of the financialization of firms with regard to the current period and the future period’s business risk, respectively. The results demonstrate that the influence of firms’ innovation intensity on both the current and future periods’ business risk is markedly negative, with coefficients of −783.9 and − 59.2, respectively. Furthermore, the coefficients are notably lower. This demonstrates that financialization can persist in mitigating business risks by enhancing operational flexibility. However, due to the intrinsic nature of financialization, the capacity to navigate future enterprise risks has been diminished.

This demonstrates that the implementation of financialization and augmented innovation intensity by pharmaceutical manufacturing firms can effectively mitigate the business risks associated with rising economic policy uncertainty.

### 5.5 Impact of economic policy uncertainty on innovation outputs

The aforementioned theory is based on the innovation input aspect of the study. However, for a comprehensive analysis of the innovation process, it is necessary to divide it into two aspects: innovation input and innovation output. In order to examine the relationship between economic policy uncertainty and innovation, this paper employs the enterprise’s patent applications in the current year as an explanatory variable in Equation 1, and performs a regression analysis. The results indicate that the coefficient of economic policy uncertainty is significantly positive at the 5% level, with a coefficient of 0. This suggests that economic policy uncertainty can facilitate firms’ innovation output. It is anticipated that an increase in economic policy uncertainty by one standard deviation (2.03) will result in an 8.26% increase in the level of innovation output by firms (0.0407 × 2.03) (see Table 14).

**Table 14:** The results of the basic regression of economic policy on innovation output.

	(1)	(2)
	PAT	PAT
LNPU	0.0511^***^	0.0407^**^
	(0.0152)	(0.0182)
LEV		0.214
		(0.399)
FCF		−0.161
		(0.532)
TQ		−0.0251
		(0.0306)
TOP1		0.0048
		(0.0072)
SIZE1		0.144
		(0.132)
GL		−0.368
		(0.493)
YX		−0.877
		(0.547)
YY		0.0345^***^
		(0.0122)
Constant	2.323^***^	−0.0480
	(0.0785)	(2.902)
*N*	1,128	1,128
*R* ^2^	0.019	0.035
Adj. *R*^2^	0.018	0.028

*p < 0.1, **p < 0.05, ***p < 0.01.

## 6 Conclusions and recommendations

As economic development progresses, the normalization of economic policy uncertainty becomes a prevalent phenomenon. The rise of economic policy uncertainty gives rise to market turbulence and increased risk, which in turn have a far-reaching impact on the macroeconomy as well as corporate behavior. The question of how to deal with the impact of economic policy uncertainty has become a key area of research. The question of how to cope with the impact of economic policy uncertainty has also become a topic of considerable debate among high-tech and high-input industries, such as pharmaceutical manufacturing enterprises. This is particularly relevant in the context of China’s pursuit of high-quality economic development and the objective of a healthy China. This paper examines the impact of economic policy uncertainty on corporate innovation in the pharmaceutical manufacturing sector. Additionally, the analysis is based on a review of Chinese A-share listed companies in the pharmaceutical manufacturing industry from 2015 to 2022. The final conclusions are as follows.

(1) An increase in economic policy uncertainty has been found to have a positive effect on the level of innovation undertaken by firms. (2) In comparison to state-owned enterprises (SOEs), non-SOEs demonstrate a greater propensity to adopt strategies that enhance innovation intensity in the context of economic policy uncertainty. (3) In the context of economic policy uncertainty, pharmaceutical manufacturing firms with management with overseas backgrounds and two jobs tend to increase the innovation intensity of their firms. (4) Further analysis indicates that rising economic policy uncertainty increases firms’ own innovation intensity by prompting them to implement financial reforms, enhance executive compensation incentives, and secure government subsidies. (5) Further analysis reveals that while firms may enhance their innovation intensity and pursue financialization, this approach can help mitigate operational risk. However, the impact is often limited. With regard to operational costs, increasing innovation investment may not fully offset the adverse effects of economic policy uncertainty. Despite implementing financialization reforms, firms may not fully insulate themselves from the immediate impact of operational costs. However, they can anticipate and adapt to external operational cost fluctuations in the future. (6) An increase in economic policy uncertainty prompts pharmaceutical firms to enhance their innovation output.

In light of the aforementioned analyses, this paper puts forth the following policy implications in conjunction with the conclusions.

Firstly, for the government, the primary issue of research is the reduction of risk due to policy uncertainty, given the rapid changes in external macroeconomic policies. In light of the distinctive attributes of the pharmaceutical manufacturing industry, the industry’s development is primarily driven by continuous innovation. The government’s role is to provide guidance through signals to investors, facilitate information flow between investors and enterprises, and attract further investment. The influx of additional investors is expected to diminish the prevailing characteristics of the pharmaceutical manufacturing industry, namely its high cycle, high investment, and low return R&D. This is anticipated to have a stabilizing effect on the market for pharmaceuticals, while also reinvigorating the dynamism and vitality of China’s drug research and development. It is incumbent upon the government to proactively disseminate efficacious policy information and to enhance the transparency, predictability, and efficacy of policy-making. The timely release of policy information via official channels serves to reduce market speculation and misunderstanding. In formulating and introducing economic policies, it is essential that the government conducts comprehensive market research, anticipates potential scenarios that may emerge following the implementation of policies, and strives to mitigate the risks that may arise. For state-owned pharmaceutical manufacturing enterprises, the introduction of appropriate incentives and penalty mechanisms can facilitate the rejuvenation of their innovative vitality. In addition, the establishment of a flatter management structure and process can enhance the enterprise’s capacity to respond rapidly and flexibly to market changes. Furthermore, the challenges posed by economic policy uncertainty can be effectively addressed through the implementation of rapid decision-making and flexible adjustment.

Secondly, for enterprises in the pharmaceutical manufacturing industry, innovation represents a fundamental basis for their activities. Enterprise financial reform is frequently only a short-term solution that cannot address the underlying issue. It may, however, introduce a ‘real to virtual’ risk for pharmaceutical manufacturing enterprises. It is incumbent upon enterprises to define their own strategic innovation direction and goals. This must be done with an in-depth understanding of market demand, combined with macroeconomic policy and corporate development planning. The establishment of different cycles of innovation strategy is necessary to guide the enterprise in the face of fluctuations in different external environments and to maintain the stability of the innovation program. It is imperative that pharmaceutical manufacturing enterprises enhance their economic resilience and effectively navigate the challenges and opportunities presented by economic policy uncertainty. It is recommended that attention be focused on the training of internal management, the establishment of a reward and punishment mechanism, the integration of innovation into corporate culture, the encouragement of employees to actively participate in innovation activities, and the formation of a favorable atmosphere for all staff to innovate. It is essential to enhance the enterprise’s innovation capabilities and accelerate technological innovation. It is essential to establish an effective risk management mechanism to identify, assess, and control the risks associated with economic policy uncertainty. In periods of elevated economic policy uncertainty, it is incumbent upon enterprises to proactively identify and pursue a multiplicity of financing channels, with a view to mitigating the financial costs and risks associated with their activities.

## 7 Limitations and future work

This paper is limited in that it focuses exclusively on the mechanism of innovation inputs, without analyzing the mechanism of innovation outputs or studying the quality of outputs. For the pharmaceutical manufacturing industry, the quality of innovation output may also be affected by certain factors. It would be beneficial for future research to investigate the impact of economic policy uncertainty on the quality of innovation output, with a view to proposing appropriate countermeasures.

## Data Availability

Publicly available datasets were analyzed in this study. This data can be found here: CSMAR.

## Footnotes

^1^The Baker team updated the economic policy uncertainty index based on the calculations of Baker (68), sourced from the economic policy uncertainty index on China at http://www.policyuncertainty.com.
